# Transcriptome analysis of periodontitis-associated fibroblasts by CAGE sequencing identified DLX5 and RUNX2 long variant as novel regulators involved in periodontitis

**DOI:** 10.1038/srep33666

**Published:** 2016-09-20

**Authors:** Masafumi Horie, Yoko Yamaguchi, Akira Saito, Takahide Nagase, Marina Lizio, Masayoshi Itoh, Hideya Kawaji, Timo Lassmann, Piero Carninci, Alistair R. R. Forrest, Yoshihide Hayashizaki, Tatsuo Suzutani, Kai Kappert, Patrick Micke, Mitsuhiro Ohshima

**Affiliations:** 1 Department of Respiratory Medicine, Graduate School of Medicine, The University of Tokyo, 7-3-1 Hongo, Bunkyo-ku, Tokyo 113-0033, Japan; 2 Division for Health Service Promotion, The University of Tokyo, 7-3-1 Hongo, Bunkyo-ku, Tokyo 113-0033, Japan; 3 Division of Genomic Technologies (DGT), RIKEN Center for Life Science Technologies, 1-7-22 Suehiro-cho, Tsurumi-ku, Yokohama, Kanagawa 230-0045, Japan; 4 Department of Biochemistry, Nihon University School of Dentistry, 1-8-13 Kanda-Surugadai, Chiyoda-ku, Tokyo 101-8310, Japan; 5 Division of Functional Morphology Dental Research Center Nihon University School of Dentistry, 1-8-13 Kanda-Surugadai, Chiyoda-ku, Tokyo 101-8310, Japan; 6 Omics Science Center, RIKEN Yokohama Institute, 1-7-22 Suehiro-cho, Tsurumi-ku, Yokohama, Kanagawa 230-0045, Japan; 7 RIKEN Preventive Medicine and Diagnosis Innovation Program, 2-1 Hirosawa, Wako, Saitama 351-0198, Japan; 8 Harry Perkins Institute of Medical Research, QEII Medical Centre and Centre for Medical Research, the University of Western Australia, Nedlands, Western Australia, Australia; 9 Department of Microbiology, Fukushima Medical University School of Medicine, 1 Hikariga-oka, Fukushima, Fukushima 960-1295, Japan; 10 Institute of Laboratory Medicine, Clinical Chemistry and Pathobiochemistry, Center for Cardiovascular Research (CCR), Charité-University Medicine Berlin, Berlin, Germany; 11 Department of Immunology, Genetics and Pathology, Akademiska Sjukhuset, Uppsala University, 751 85 Uppsala, Sweden; 12 Department of Biochemistry, Ohu University School of Pharmaceutical Sciences, Misumido 31–1, Tomitamachi, Koriyama, Fukushima 963–8611, Japan

## Abstract

Periodontitis is affecting over half of the adult population, and represents a major public health problem. Previously, we isolated a subset of gingival fibroblasts (GFs) from periodontitis patients, designated as periodontitis-associated fibroblasts (PAFs), which were highly capable of collagen degradation. To elucidate their molecular profiles, GFs isolated form healthy and periodontitis-affected gingival tissues were analyzed by CAGE-seq and integrated with the FANTOM5 atlas. GFs from healthy gingival tissues displayed distinctive patterns of CAGE profiles as compared to fibroblasts from other organ sites and characterized by specific expression of developmentally important transcription factors such as *BARX1*, *PAX9*, *LHX8*, and *DLX5*. In addition, a novel long non-coding RNA associated with *LHX8* was described. Furthermore, we identified *DLX5* regulating expression of the long variant of *RUNX2* transcript, which was specifically active in GFs but not in their periodontitis-affected counterparts. Knockdown of these factors in GFs resulted in altered expression of extracellular matrix (ECM) components. These results indicate activation of *DLX5* and *RUNX2* via its distal promoter represents a unique feature of GFs, and is important for ECM regulation. Down-regulation of these transcription factors in PAFs could be associated with their property to degrade collagen, which may impact on the process of periodontitis.

Periodontitis is characterized by gingival inflammation accompanied by loss of supportive connective tissues for the tooth, resulting in impaired attachment of the periodontal ligament to the cementum. Periodontitis is one of the most common diseases in humans that affects over half of the adult population. Tooth loss caused by periodontitis is associated with masticatory dysfunction and poor nutritional status, and the medical cost for periodontitis and related diseases is an escalating burden to the healthcare economy^1^. The goal of conventional treatments for periodontitis has been to control the infection of gingival tissues; however, many cases of periodontitis are resistant or refractory to antimicrobial therapies including antibiotics, antimicrobial mouth rinse, and removal of dental plaque^2^.

Beyond the conventional view of periodontitis as an infectious disease, an increasing number of studies focus on the aberration of cellular responses in the periodontitis-affected gingival tissues. Several studies have performed comprehensive transcriptome analyses of the gingival tissues of periodontitis patients^3^,^4^,^5^, which improved our understanding of the molecular mechanisms underlying the pathogenesis of periodontitis^3^. However, these analyses provided little information on the cellular level, and it remained unknown which definite cell type is critical for the altered gene expression profiles in periodontitis.

A hallmark of periodontitis is degradation of extracellular matrices (ECM), such as collagen, between the tooth root and the alveolar bone. In various organs, the fibroblast is a central player to form the structural framework and control tissue repair by regulating ECM turnover and remodeling. Gingival fibroblasts play important roles not only in the homeostasis of gingival tissue architecture but also in the pathogenesis of periodontitis^2^. Recently, we isolated and characterized a subset of gingival fibroblasts derived from periodontitis-affected patients that were designated as periodontitis-associated fibroblasts (PAFs), and demonstrated that they were highly capable of collagen degradation^6^,^7^.

Preventing ECM degradation in supportive connective tissues for the tooth seems a straightforward therapeutic approach for periodontitis. As a proof of concept, we have previously demonstrated that targeting PAFs by inhibition of key signaling pathways, i.e., transforming growth factor-β (TGF-β) and vascular endothelial growth factor (VEGF), successfully protected PAF-mediated degradation of collagen in experimental models of periodontitis^6^,^7^. Obviously, it is important to elucidate the PAF phenotype from diagnostic and therapeutic viewpoints; however, the molecular mechanisms how PAFs appear and contribute to ECM degradation in the periodontitis tissues are largely unknown.

To further characterize and dissect the molecular repertoire of PAFs we took advantage of the ongoing project of the Functional annotation of the mammalian genome (FANTOM) 5. FANTOM 5 is an international research consortium that has released transcriptome data for about one thousand human samples including cell lines, primary cells, and tissues, using the cap analysis of gene expression (CAGE) technology, which captures the 5′ end of capped transcripts and sequences around 27 base pairs^8^. CAGE analysis allowed us to map transcription start sites and promoter regions both for coding and non-coding transcripts across the whole genome, simultaneously providing a quantitative measure of transcriptional activity among the numerous FANTOM5 samples^9^,^10^.

As part of the FANTOM5 project, we performed CAGE analyses on primary cultured human gingival fibroblasts, periodontal ligament fibroblasts, gingival epithelial cells, and epithelial cell rests of Malassez^11^. Furthermore, CAGE profiles of PAFs were also examined together with patient-matched gingival fibroblasts derived from healthy gingival tissues, helping us to identify specific molecular features and novel markers that are potentially of functional relevance^7^.

First we investigated if gingival fibroblasts have molecular characteristics distinct from fibroblasts derived from other organs as suggested by a previous report^12^. Next we compared CAGE profiles of PAFs and normal counterparts to examine gene expression patterns related to the PAF phenotype. Through these analyses we discovered that the expression of Distal-Less Homeobox 5 (*DLX5*) and Runt-related transcription factor 2 (*RUNX2*) in its long transcript form is specific for gingival fibroblasts, and is virtually lost in PAFs. Further transcriptome analyses revealed that these identified genes are involved in the regulation of ECM. Our findings supported the hypothesis that altered molecular signals mediated by *DLX5* and *RUNX2* long form are associated with the aggressive phenotype of PAFs and degradation of ECM.

This work is part of the FANTOM5 project. Data downloads, genomic tools, and co-published manuscripts are summarized online at http://fantom.gsc.riken.jp/5/.

## Result

### Distinctive CAGE profiles of gingival fibroblasts

A previous study has shown that human fibroblasts derived from different sites of the body display differential gene expression patterns^12^, highlighting the heterogeneity of fibroblasts. In order to define transcriptional profiles characteristic for gingival fibroblasts (GFs) in comparison with other fibroblasts, the CAGE data of totally 45 primary cultured fibroblasts derived from different anatomic sites were extracted from the FANTOM5 database (Supplementary Table S1). These samples included 6 gingival fibroblast (GF) cultures (GF1, GF2, and GF3 were commercially available; GF4, GF5, and GF6 were established and provided by us) and six periodontal ligament fibroblast (PLF) cultures (PLF1, PLF2, and PLF3 were commercially available; PLF4, PLF5, and PLF6 were established and provided by us).

**Figure 1:**
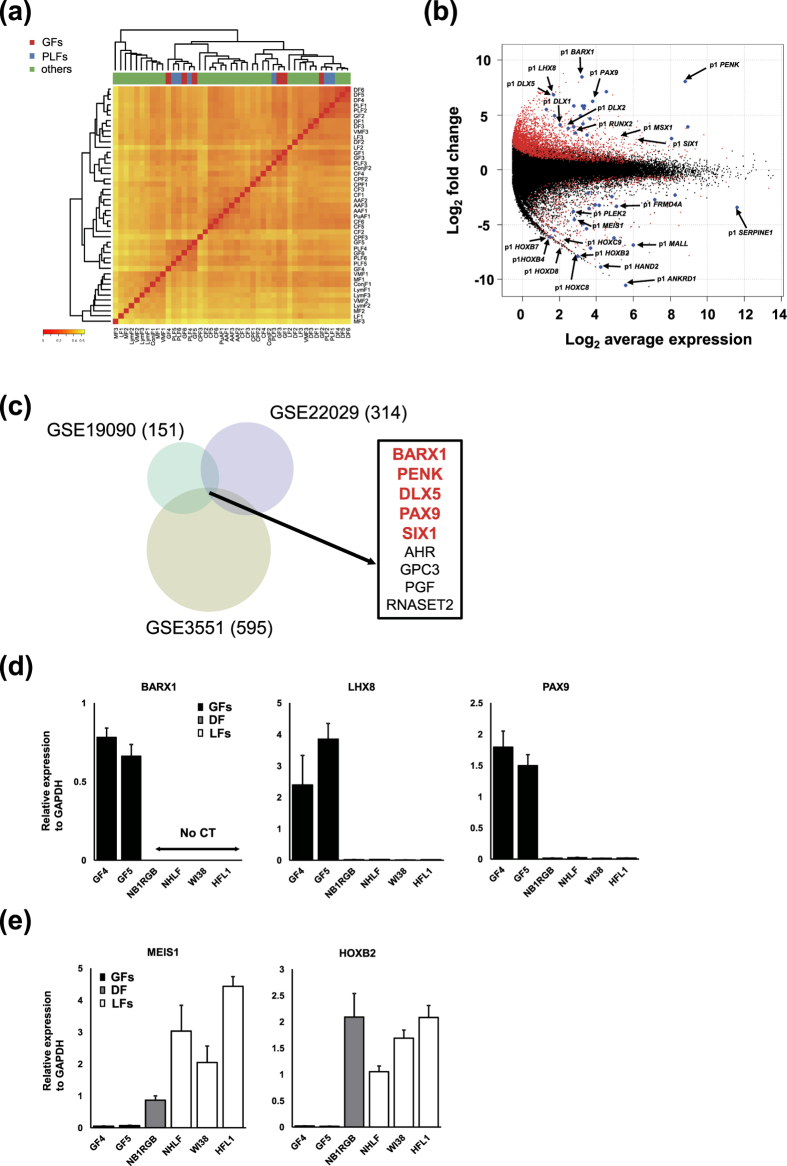
(**a**) Hierarchical clustering analysis with all DPIs of 6 gingival fibroblasts (GFs) (red), 6 periodontal ligament fibroblasts (PLFs) (blue), and 33 other fibroblasts derived from different anatomic sites (green) by Ward’s method. The details of fibroblasts are described in Supplementary Table S1. Red to yellow color gradient of heatmap represents the degree of correlation of the indicated cell pair. (**b**) MA plot showing expression differences between GFs and 33 other fibroblasts. Red marks indicate promoters with significantly differential expression defined by false discovery rate (FDR) < 0.05, and blue marks indicate the genes with highly differential expression after sorting by FDR. The *x*-axis represents expression strength of a gene measured by CAGE tag counts and shown as average log_2_ counts per million. The *y*-axis represents fold changes of gene expression shown as log_2_ values. Positive fold changes indicate higher expression in GFs. (**c**) Venn diagram of genes with higher expression in GFs compared to other fibroblasts. The results from 3 microarray datasets were merged (GSE3551, GSE19090, GSE22029). The number of genes with higher expression in GFs (log_2_ fold change >1, FDR < 0.05) is indicated in parenthesis. The indicated 9 genes were common in the 3 datasets. Among them, 5 genes highlighted in red were also enriched in GFs as determined by CAGE profiling (**b**). (**d**) RT-qPCR for *BARX1*, *LHX8*, and *PAX9* in GFs (black bars: GF4 and GF5), dermal fibroblasts (DF, grey bar: NB1RGB), and lung fibroblasts (LFs, white bars: NHLF, WI38, and HFL1). The expression of each gene was normalized to that of *GAPDH*. Bars represent mean ± SD. No CT indicates the failure to calculate Ct (cycle threshold) values due to undetectable expression. (**e**) RT-qPCR for *MEIS1* and *HOXB2* in GFs, DF, and LFs. The data is presented as in (**d**).

Unsupervised hierarchical clustering with all DPIs revealed that primary cultures of GF and PLF established in our institute were grouped together (Fig. 1(a)). On the other hand, the commercially available GFs and PLFs were grouped into two separate clusters. Comparison of CAGE profiles between GFs and PLFs did not show any significant difference (data not shown), indicating GFs and PLFs have similar CAGE profiles.

CAGE peaks represent transcription start sites (TSSs), and their profiling is useful to identify the locations and usage levels of TSSs. We can thereby evaluate expression levels of transcripts and promoter activities across the whole genome. To describe the CAGE profiles of GFs, CAGE tag counts of all 6 GFs were compared to those of all other 33 fibroblasts except PLFs. In total 3633 CAGE-defined promoters showed significantly higher expression, while 514 promoters displayed lower expression (FDR < 0.05, Supplementary Table S3). First we aimed to investigate gene-wise expression differences and focused on CAGE-defined promoters annotated as peak 1 (p1) which shows the highest expression among alternative promoters for the same gene. As a result, as much as 195 and 109 p1 promoters showed higher and lower expression, respectively, by the strict criteria as follows; log_2_ fold change >2 or <−2, log_2_ counts per million >1, and matching single gene annotation (Supplementary Table S4). Differential gene expression was further illustrated in MA-plot, which showed the relationship between the magnitude of differential expression (fold change) and average expression level of each gene of 39 all fibroblasts (Fig. 1(b)). To predict the function of differentially expressed genes in GFs, GO analysis was performed. The predicted functions of the 195 genes enriched in GFs were related to organ development and many genes represented transcription factors (Supplementary Table S5, Supplementary Figure S1(a)).

Notably, highly expressed genes in GFs included BarH-Like Homeobox 1 (*BARX1*), Paired Box 9 (*PAX9*), Lim Homeobox 8 (*LHX8*), Distal-Less Homeobox 1 (*DLX1*), *DLX2*, *DLX5*, and Msh Homeobox 1 (*MSX1*), all of which have been shown as master regulators of mesenchymal cells during the tooth development^13^. These findings may indicate that adult GFs maintain key transcriptional features involved in odontogenesis as ‘positional memory’ and that these genes are still of relevance to maintain tissue homeostasis^12^. Since regulatory mechanisms of tissue repair and regeneration have similarities with those for development and organogenesis, our findings suggested the potential roles of key transcription factors in the pathogenesis of periodontitis.

In analogy, many genes that showed lower expression in GFs were transcription factors. The functions of the 109 genes with lower expression in GFs predicted by GO analysis were also associated with organ development (Supplementary Table S6, Supplementary Figure S1(b)). Interestingly, GFs showed extremely low expression levels of homeotic (HOX) genes crucial for body positioning during development, such as *HOXB2*, *HOXB4*, *HOXB7*, *HOXC8*, *HOXC9*, and *HOXD8*, as compared to other fibroblasts^14^. Expression profiling of all HOX genes showed that GFs do not express any Hox gene while a subset of HOX genes are expressed in other fibroblasts (Supplementary Figure S2(a)). These findings further supported the notion that GFs represent a unique cell population distinct from other fibroblasts.

To validate the findings based on the FANTOM5 dataset, 3 microarray datasets which evaluated various fibroblasts including GFs were analyzed focusing on GFs^12^,^15^,^16^. Differential gene expression was determined following the criteria, FDR < 0.05 and log_2_ fold change >1 or <−1. Through the analyses on GSE3551 (4 GFs vs 46 others), GSE19090 (6 GFs vs 33 others), and GSE22029 (8 GFs vs 8 dermal fibroblasts) datasets, 595, 151, and 314 genes were found to be significantly enriched in GFs, respectively (Supplementary Table S7). Heatmaps of the top 150 genes with significantly differential expression between GFs and other fibroblasts are shown in Supplementary Figure S2(b). Furthermore, 9 genes showed higher expression in GFs commonly across these 3 datasets (Fig. 1(c)). Among them, 5 genes also showed significantly higher expression in GFs in the FANOM5 database (Supplementary Table S4). These robust GF-specific genes were *BARX1*, Proenkephalin (*PENK*), *DLX5*, *PAX9*, and SIX Homeobox 1 (*SIX1*) (Fig. 1(c)).

Among the genes that showed specific expression in GFs, we selected several genes that encode key transcription factors for RT-qPCR validation. Consistent with the finding of CAGE profiles, *BARX1*, *LHX8*, and *PAX9* were confirmed to be specifically expressed in GFs as compared with lung fibroblasts (LFs) and dermal fibroblasts (DF) (Fig. 1(d)). In contrast, *HOXB2* and Meis Homeobox 1 (*MEIS1*), both underexpressed in the FANTOM5 dataset, were not detected in GFs while expressed in LFs and DF (Fig. 1(e)).

### CAGE revealed activation of alternative promoter of RUNX2 in GFs

**Figure 2:**
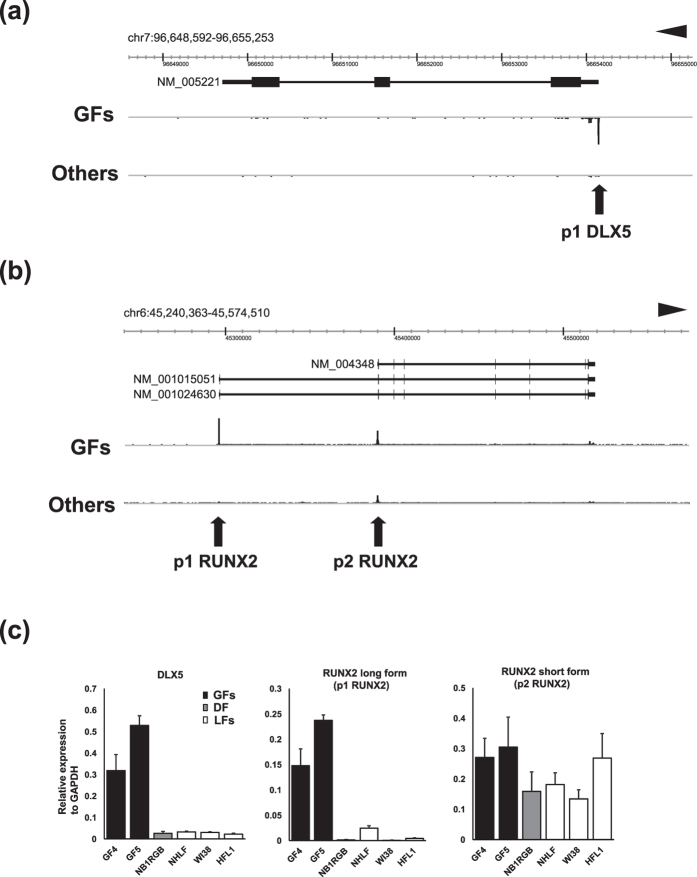
(**a**) CAGE peaks of 6 GFs and 33 other fibroblasts visualized by the ZENBU browser. Genomic coordinate and transcript of *DLX5* registered in RefSeq (NM_005221) are shown on the top. CAGE peak for p1 DLX5 is prominent in GFs in contrast to 33 other fibroblasts. (**b**) CAGE peaks of 6 GFs and 33 other fibroblasts visualized by the ZENBU browser. Genomic coordinate and 3 protein coding transcript variants of *RUNX2* registered in RefSeq (NM_004348, NM_001015051, and NM_001024630) are shown on the top. CAGE peaks for both p1 RUNX2 and p2 RUNX2 are detected in GFs while other fibroblasts show a dominant peak for p2 RUNX2. (**c**) RT-qPCR for *DLX5*, *RUNX2* long form transcribed from p1 RUNX2 promoter, and *RUNX2* short form transcribed from p2 RUNX2 promoter. Primers for *RUNX2* long form were designed so that one half hybridizes to the second exon and the other half to the third exon. Primers for *RUNX2* short form were designed to amplify the isoform-specific sequence of the first exon. The expression of each gene was normalized to that of *GAPDH*. Bars represent mean ± SD.

CAGE analysis provides definitive information of alternative promoters. In addition to comparative analyses of gene-wise expression (Supplementary Table S4), we also analyzed promoter-level expression differences between GFs and other fibroblasts to identify alternative promoters specific for GFs (Supplementary Table S3). We noted different expression patterns of transcript variants for *RUNX2*, a master regulator of osteoblast differentiation and bone formation^17^. Importantly, it has previously been shown that *DLX5* specifically transactivates the *RUNX2* distal promoter to confer cell type-specific expression of *RUNX2* isoforms^18^. Since *DLX5* is a robust GF-specific gene identified by our comparative analyses on the FANTOM5 CAGE profiles as well as 3 different microarray datasets (Fig. 1(b,c)), we further studied the associations between *DLX5* and *RUNX2* alternative promoters in GFs and 33 other fibroblasts. We used the FANTOM5 ZENBU genome browser for CAGE validation^19^, and confirmed that the CAGE peak of p1 DLX5 appears specifically in GFs (Fig. 2(a)).

Among the transcript variants of *RUNX2*, long forms (NM_001015051 and NM_001024630) are transcribed from distal p1 promoter while a short form (NM_004348) uses proximal p2 promoter (Fig. 2(b)). We found that p1 RUNX2 was highly expressed in GFs indicating that *RUNX2* long form is specifically transcribed in GFs. In contrast, *RUNX2* short form, transcribed from p2 RUNX2, appeared ubiquitously expressed both in GFs and other fibroblasts (Fig. 2(b)).

To validate these findings, RT-qPCR for *DLX5*, *RUNX2* long and short forms were performed. Primers for *RUNX2* long form were designed in a way that one half hybridizes to the second exon and the other half to the third exon. Primers for *RUNX2* short form were designed to amplify the isoform-specific sequence of the first exon. Consistent with the finding of CAGE analysis, *RUNX2* long form was specifically detected in GFs whereas the expression of *RUNX2* short form was confirmed both in GFs and other fibroblasts (Fig. 2(c)).

### CAGE identified novel GF-specific non-coding RNAs

Accumulating evidence demonstrates that more than 60% of the genome is transcribed as RNA, and most of the transcripts are non-coding RNAs (ncRNAs)^20^,^21^,^22^. The CAGE technique used in the FANTOM5 project allows the analysis of both coding and non-coding transcripts. We also compared CAGE peaks for ncRNAs between GFs and other fibroblasts (FDR < 0.05, log_2_ fold change >2 or <−2, and log_2_ counts per million >1), and found that 34 and 16 ncRNAs showed significantly higher and lower expression in GFs, respectively (Supplementary Table S8).

**Figure 3:**
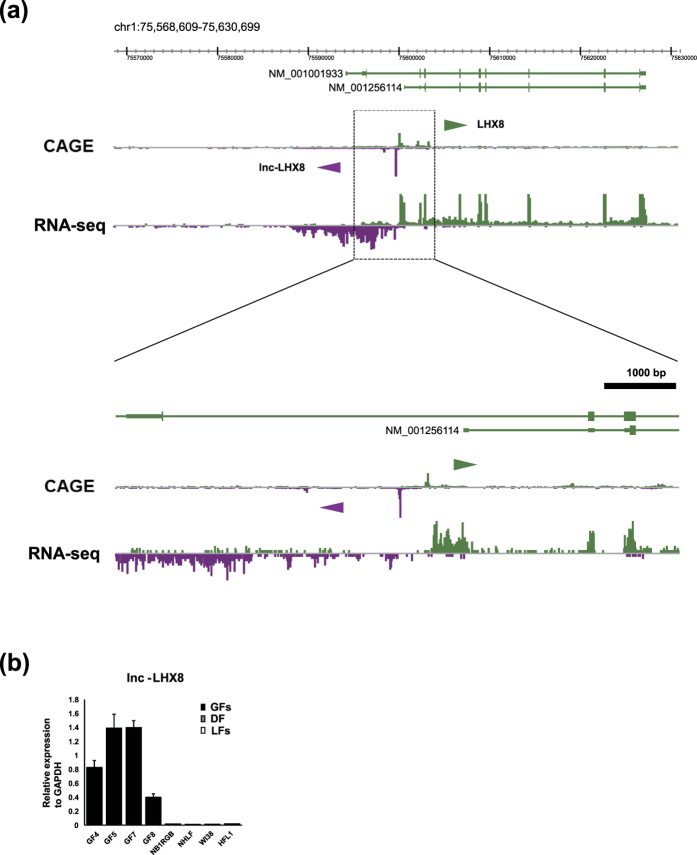
(**a**) RNA-seq of GF8 (lower panel) and CAGE peak of GF4 (upper panel). LHX8 and neighboring ncRNAs which are specifically expressed in normal GFs were visualized by the ZENBU browser. (**b**) RT-qPCR for *lnc-LHX8* in GFs (GF4, GF5, GF7, and GF8) and other fibroblasts (DF and LFs). The expression was normalized to that of *GAPDH*. Bars represent mean ± SD.

The ncRNA with TSS at chr1:75599683-75599699 on the minus strand showed the most specific expression in GFs (FDR = 1.38 × 10^−82^). Notably, this CAGE peak is located within 1,000 bp of the TSS of *LHX8* (Fig. 3(a), Supplementary Table S8), a GF-specific coding gene identified by our comparative analysis. The colocalization and parallel expression patterns in GFs suggested that transcription of this ncRNA might be associated with the state of chromatin and *LHX8* gene expression (Supplementary Figure S3).

To supplement the CAGE analysis on TSSs with sequence information, we further performed RNA-seq. Combined analyses of RNA-seq and CAGE data revealed that the newly identified TSS is for long non-coding RNAs (lncRNAs) that share the first exon and have several splicing variants. We designated these transcripts as *lnc-LHX8* (Fig. 3(a)). In order to validate the distinctive expression of *lnc-LHX8* in GFs, specific primers for *lnc-LHX8* were designed to amplify the first exon, and RT-qPCR was performed. Consistent with the findings of CAGE and RNA-seq analyses, transcripts for *lnc-LHX8* were exclusively detected in GFs, indicating the highly specific expression (Fig. 3(b)).

Collectively, analyses on the CAGE profiles of GFs as compared to other fibroblasts delineated the unique transcriptional patterns of coding genes, alternative promoters, and ncRNAs.

### Distinctive CAGE profiles of PAFs

**Figure 4:**
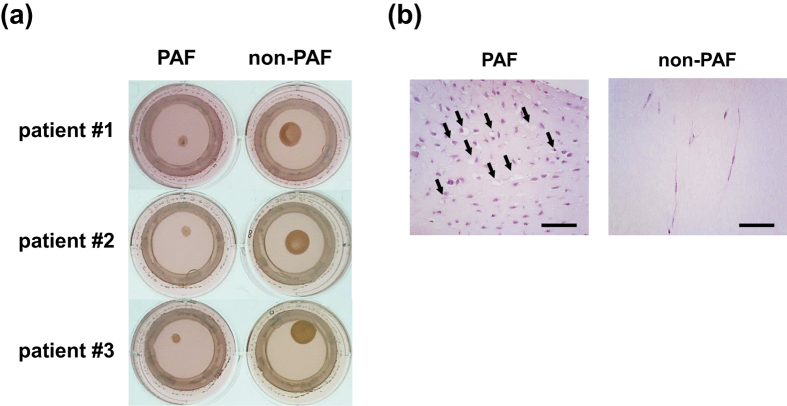
(**a**) Collagen gels embedded with patient-matched periodontitis-associated fibroblasts (PAFs) or non-PAFs derived from healthy gingival tissues. Gingival epithelial cells, which are not patient-matched, were co-cultured on the surface of each gel for 5 d. Representative pictures of collagen gels cultured separately under air-liquid interface conditions. The brown spot in each well is the remaining collagen gel. The size of collagen gel embedded with PAFs was smaller than that seen in the non-PAF treatment. (**b**) Representative pictures of hematoxylin and eosin (H&E) staining of collagen gels embedded with PAFs or non-PAFs. Arrows indicate degradation of collagen gel matrix adjacent to PAFs. Bar represents 25 μm.

In the following analyses, we focused on gingival fibroblasts derived from patients suffering from periodontitis. Three pairs of patient-matched gingival fibroblasts were isolated from periodontitis-affected and healthy gingival tissues, and designated as periodontitis-associated fibroblasts (PAFs) and non-periodontitis-associated fibroblasts (non-PAFs). Consistent with our previous report^7^, PAFs induced collagen degradation more strongly than non-PAFs in an established three-dimensional co-culture model of periodontitis (Fig. 4(a)). H&E staining of collagen gels showed degradation of collagen matrix adjacent to PAFs (Fig. 4(b)). To explore transcriptional profiles underlying the functional differences between PAFs and non-PAFs, CAGE analysis was performed using the RNA samples of these primary cultured fibroblasts derived from periodontitis patients.

**Figure 5:**
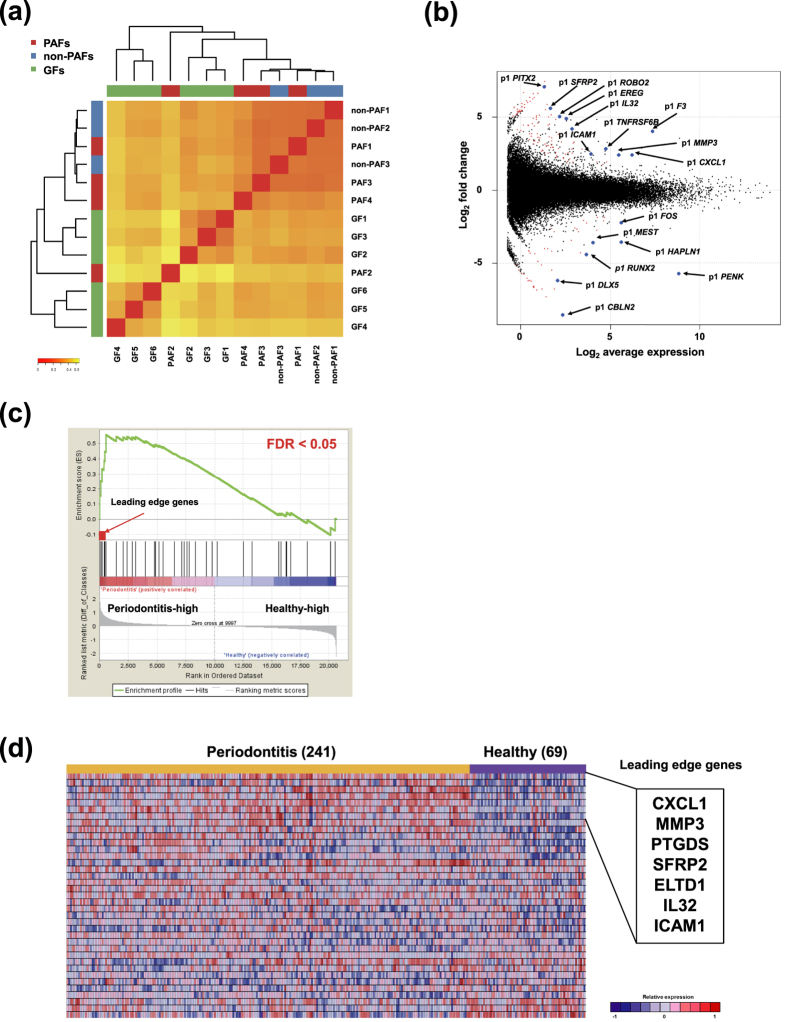
(**a**) Hierarchical clustering analysis with all DPIs of PAFs (PAF1, PAF2, PAF3, and PAF4), non-PAFs (non-PAF1, non-PAF2, and non-PAF3), and control GFs (GF1, GF2, GF3, GF4, GF5, and GF6) by Ward method. Red to yellow color gradient of heatmap represents the degree of correlation of the indicated cell pair. (**b**) MA plot showing expression differences between 3 patient-matched PAFs and non-PAFs. Red marks indicate genes (p1 promoters) with significantly differential expression defined by false discovery rate (FDR) < 0.1, and blue marks indicate the genes with highly differential expression after sorting by FDR. The *x*-axis represents expression strength of a gene measured by CAGE tag counts and shown as average log_2_ counts per million. The *y*-axis represents fold changes of gene expression shown as log_2_ values. Positive fold changes indicate higher expression in PAFs. (**c**) Gene set enrichment analysis (GSEA) reveals the enrichment of 48 “PAF-related genes” in periodontitis tissues (n = 241) compared to healthy gingival tissues (n = 69) from GSE16134. The genes that correlated with the periodontitis and healthy phenotypes are indicated on the left (‘Periodontitis-high’) and right (‘Healthy-high’), respectively. The seven leading edge genes are indicated with an arrow. (**d**) Heatmap representing the relative expression levels of 37 “PAF-related genes” which could be annotated both in the CAGE and GSE16134 datasets. The seven leading edge genes identified in (**c**) are highlighted. Red to blue color gradient of heatmap represents the relative gene expression levels.

Hierarchical clustering with all DPIs of PAFs, non-PAFs, and normal GFs revealed that both PAFs and non-PAFs tended to be grouped into the same cluster (Fig. 5(a)). Next we explored promoter-level expression differences between PAFs and non-PAFs, and identified 112 up-regulated and 46 down-regulated promoters, including alternative promoters for the same genes (FDR < 0.1) (Fig. 5(b), Supplementary Table S9). Analyses of transcriptional differences at gene expression level revealed 48 up-regulated and 19 down-regulated coding genes in PAFs (Supplementary Table S10).

GO analysis showed that up-regulated genes in PAFs were involved in signal transduction such as interleukin 32 (*IL32*), chemokine (C-X-C motif) ligand 1 (*CXCL1*), epiregulin (*EREG*), and secreted frizzled-related protein 2 (*SFRP2*), regulation of immune effector process such as complement component 3 (*C3*), and dipeptidyl peptidase-4 (*DPP4*), and cell adhesion such as intercellular adhesion molecule 1 (*ICAM1*), cadherin 18 (*CDH18*), and claudin 1 (*CLDN1*) (Supplementary Table S11, Supplementary Figure S4).

We supplemented the CAGE analysis of PAFs with publicly available microarray datasets from 241 periodontitis and 69 healthy gingival tissue samples (GSE16134)^3^. The 48 up-regulated genes from the GAGE analysis of PAFs (Supplementary Table S10, hereafter referred to as PAF-related genes) were also significantly enriched in the periodontitis tissues (FDR < 0.05) (Fig. 5(c)). Among them, seven genes with the highest association were *CXCL1*, matrix metalloproteinase-3 (*MMP3*), prostaglandin D2 synthase (*PTGDS*), *SFRP2*, EGF-TM7-Latrophilin-Related protein (*ELTD1*), *IL32*, and *ICAM1*. The heatmap of 37 genes which could be annotated both in the CAGE and GSE16134 datasets are shown in Fig. 5(d).

### CAGE revealed inactivation of DLX5 and RUNX2 distal promoter in PAFs

**Figure 6:**
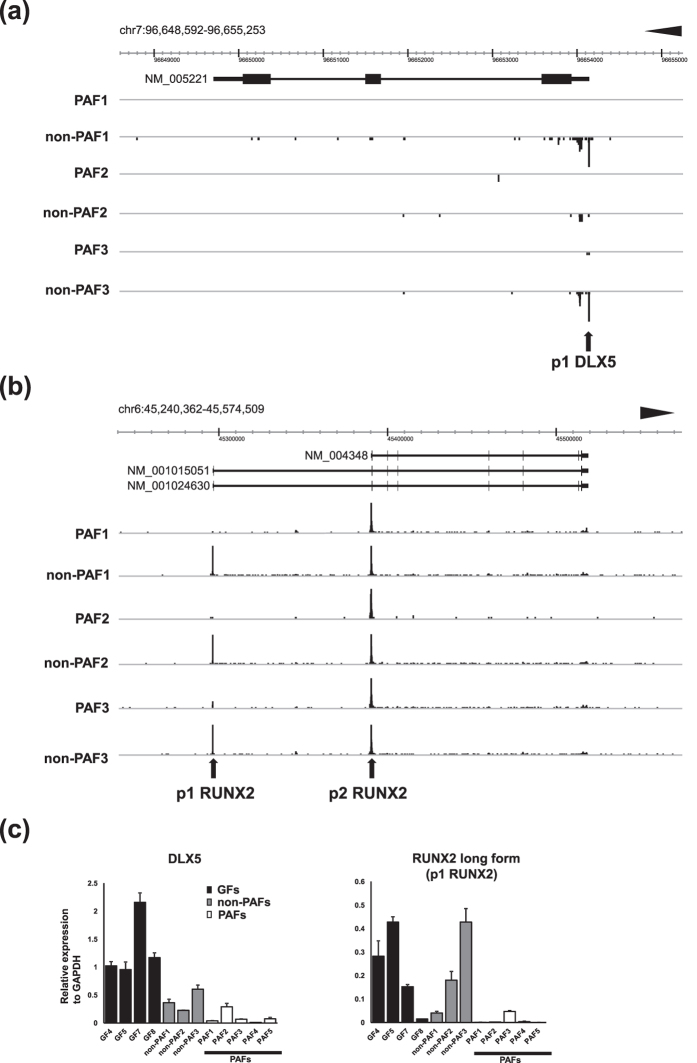
(**a**) CAGE peaks of patient-matched 3 PAFs and 3 non-PAFs visualized by the ZENBU browser. Genomic coordinate and transcript of *DLX5* registered in RefSeq (NM_005221) are shown on the top. CAGE peak for p1 DLX5 was observed in non-PAFs while absent in PAFs. (**b**) CAGE peaks of patient-matched 3 PAFs and 3 non-PAFs visualized by the ZENBU browser. Genomic coordinate and 3 protein coding transcript variants of *RUNX2* registered in RefSeq (NM_004348, NM_001015051, and NM_001024630) are shown on the top. CAGE peaks for both p1 RUNX2 and p2 RUNX2 are detected in non-PAFs as observed in control GFs (Fig. 2(b)) while PAFs show a dominant peak for p2 RUNX2. (**c**) RT-qPCR for *DLX5* and *RUNX2* long form transcribed from p1 RUNX2 promoter. The expression of each gene was normalized to that of *GAPDH*. Bars represent mean ± SD. The expression levels were examined in 4 control GFs (GF4, GF5, GF7, and GF8), 3 patient-matched non-PAFs (non-PAF1, non-PAF2, and non-PAF3) and PAFs (PAF1, PAF2, and PAF3), and 2 additional PAFs (PAF4 and PAF5). Each expression was normalized to that of *GAPDH*. Bars represent mean ± SD.

Next we analyzed the 46 down-regulated promoters in PAFs (Supplementary Table S9). Remarkably, 12 out of 46 down-regulated promoters were listed as those specifically up-regulated in GFs (Supplementary Table S3), which were p1 DLX5, p2 DLX5, p1 RUNX2, p24 RUNX2, p1 PENK, p3 PENK, p1 SYTL2, p2 COL10A1, p2 LAMP5, p2 PLEKHA5, p1 CBLN2, and p chr16:86532148 - 86532166-. Among 19 down-regulated genes in PAFs (Supplementary Table S10), 4 genes (*DLX5*, *RUNX2*, *PENK*, and *SYTL2*) were listed as those highly expressed in GFs (Supplementary Table S4), indicating that the GF-specific transcriptional pattern is modulated in PAFs. The detailed comparative analysis of the CAGE peaks between PAFs and non-PAFs confirmed the loss of GF-specific promoters, p1 DLX5 (Fig. 6(a), Supplementary Figure S5(a)) and p1 RUNX2 in PAFs (Fig. 6(b), Supplementary Figure S5(a)). Meanwhile, the expression of *RUNX2* short form (p2 RUNX2) was not different between PAF and non-PAFs (Fig. 6(b), Supplementary Figure S5(a)). Although there were some variations in expression due to the heterogeneity of primarny cultured GFs, we confirmed the differential expression of *DLX5* and *RUNX2* long form by RT-qPCR in 3 pairs of patient-matched PAFs and non-PAFs, additional two independent PAFs, and 4 normal GFs (Fig. 6(c)). In contrast, *RUNX2* short form transcribed from p2 RUNX2 did not show clear differences among these fibroblasts (Supplementary Figure S5(b)).

A previous report showed that DLX5 specifically transactivates the RUNX2 distal promoter, which subsequently regulates osteoblast differentiation^18^. Our findings suggested that DLX5 and RUNX2 long form are highly expressed in GFs similar to osteoblasts, and these promoter activities are lost in PAFs. To assess the similarities in terms of transcriptional profiles among GFs, PLFs, PAFs, non-PAFs, and osteoblasts, we performed unsupervised hierarchical clustering of CAGE profiles of these cell types (Supplementary Figure S6(a)). Osteoblasts and gingival fibroblasts were divided into distinct clusters, implying that transcriptional profiles between GFs and osteoblasts are largely different despite that DLX5 and RUNX2 distal promoters are preferentially activated in both cell types (Supplementary Figure S6(b)). Collectively, analyses on the CAGE data of PAFs highlighted the specific inactivation of *DLX5* and *RUNX2* distal promoters, which prompted us to explore the functional significance of these altered promoter activities.

### Functional roles of DLX5 and p1 RUNX2 in gingival fibroblast

**Figure 7:**
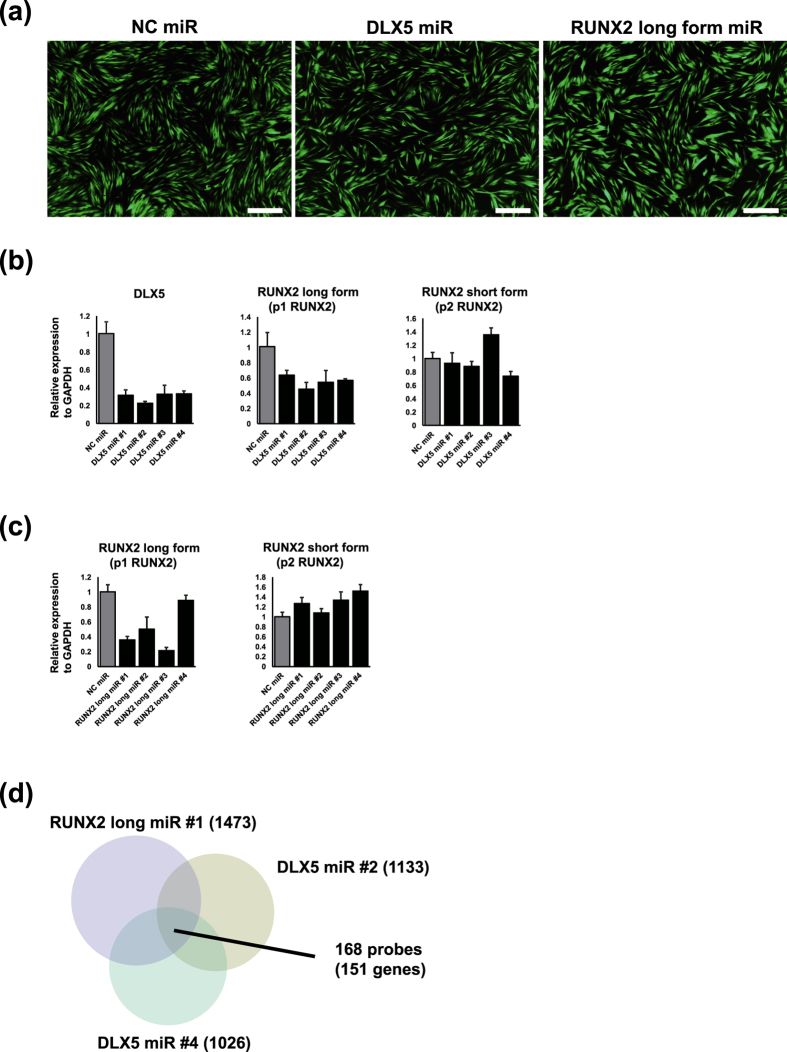
(**a**) Control GF (GF4) was infected with lentiviruses carrying artificial miRNAs together with EmGFP. Over 99% of lentivirus-infected cells were positive for EmGFP that indicate highly efficient transduction of artificial miRNAs. Control cells were transduced with negative control miRNA (NC miR). For knockdown of *DLX5* and *RUNX2* long form, 4 different miRNA sequences for each transcript were designed (DLX5 miR #1 to #4, and RUNX2 long miR #1 to #4). The pictures of fluorescence microscopy for GF4 infected with lentiviruses carrying NC miR, DLX5 miR #2, and RUNX2 long miR #1 are shown. Scale bar indicates 200 μm. (**b**) RT-qPCR for *DLX5*, *RUNX2* long form transcribed from p1 RUNX2 promoter, and *RUNX2* short form transcribed from p2 RUNX2 promoter. RNA was collected 5 d after lentiviral infection. The expression of each gene was normalized to that of *GAPDH*. Bars represent mean ± SD. (**c**) RT-qPCR for *RUNX2* long form transcribed from p1 RUNX2 promoter and *RUNX2* short form transcribed from p2 RUNX2 promoter. RNA was collected 5 d after lentiviral infection. The expression of each gene was normalized to that of *GAPDH*. Bars represent mean ± SD. (**d**) Venn diagram of down-regulated genes (fold change <0.5) by knockdown of DLX5 miR #2, #4 and RUXN2 long miR #1.

The functional roles of *DLX5* and *RUNX2* long form were evaluated by knockdown experiments using microRNAs targeting *DLX5* and *RUNX2* long form (Supplementary Table S2). Normal gingival fibroblast, GF4, was selected for these experiments because it showed abundant expression of both genes (Fig. 6(c)). Efficient transduction (over 99%) was confirmed by detecting EmGFP fluorescence, and obvious changes in cell morphology or viability were not observed following lentiviral infection (Fig. 7(a)).

RT-qPCR revealed that *DLX5* knockdown led to the down-regulation of *RUNX2* long form, while the expression of *RUNX2* short form was not influenced (Fig. 7(b)), suggesting that *DLX5* preferentially transactivates the *RUNX2* distal promoter as reported previously^18^. We could also selectively knockdown *RUNX2* long form, and importantly, the expression of *RUNX2* short form was not largely influenced by silencing of *RUNX2* long form (Fig. 7(c)).

To explore the functional roles of *DLX5* and *RUNX2* distal promoters, comparative microarray analyses were carried out in GF4 following knockdown of *DLX5* and *RUNX2* long form. Affymetrix GeneChip^®^ Human Genome U133 Plus 2.0 array was used, which contains six probes (216994_s_at, 221282_x_at, 221283_at, 232231_at, 236858_s_at, and 236859_at) for *RUNX2*. Among them, target sequence of 236859_at is in the first exon and 5′UTR, which is specific for *RUNX2* long form transcribed from p1 RUNX2. Microarray results confirmed specific knockdown of *RUNX2* long form as observed in RT-qPCR experiments. Transcriptional profiling of GF4 transduced with DLX5 miR #2 (Supplementary Table S12), DLX5 miR #4 (Supplementary Table S13), and RUNX2 miR #1 (Supplementary Table S14) revealed that 1133, 1026, and 1473 probes were down-regulated, respectively. Comparison between DLX5 miR #2 and DLX5 miR #4 showed 337 common probes, and surprisingly, as much as 168 probes (151 genes) were commonly down-regulated by DLX5 miR #2, DLX5 miR #4, and RUNX2 miR #1 (Fig. 7(d), Supplementary Table S15), indicating that transcriptional regulations by *DLX5* are largely mediated by the induction of *RUNX2* long form. GO analysis revealed that these common genes are predominantly involved in ECM organization and cell adhesion, such as collagen (*COL14A1*, *COL15A1*, *COL5A1*, and *COL8A2*), elastin (*ELN*), matrilin 3 (*MATN3*), dermatopontin (*DPT*), fibrillin 2 (*FBN2*) and vascular cell adhesion molecule 1 (*VCAM1*) (Supplementary Table S15).

Taken together, activation of *DLX5* and *RUNX2* distal promoters represents the unique feature of GFs, and is important for ECM regulation. Down-regulation of these transcription factors in PAFs is likely to modify the expression of ECM proteins and cell adhesion, which might be involved in the pathogenesis of periodontitis.

## Discussion

This study evaluates the transcriptional profiles of GFs and PAFs as their pathogenic counterparts, by CAGE sequencing as part of the FANTOM5 project. We compared GFs to human fibroblasts from other tissues, which revealed the transcriptional profiles of GFs for coding genes, alternative promoters, and ncRNAs. To our knowledge, this is the first comprehensive characterization of the transcriptome characteristic for GFs.

Fibroblasts are ubiquitous mesenchymal cells that play important roles in development, tissue repair, and various diseases such as fibrosing diseases and cancers. In periodontitis, fibroblasts critically contribute to its pathology by producing inflammatory cytokines and altering gingival tissue architectures^7^. It has been reported that fibroblasts from different anatomic sites have characteristic gene expression patterns^12^,^23^. However, transcriptional features of GFs distinct from other fibroblasts have not been investigated and remained largely unknown.

In the present study, we discovered that a subset of transcription factors such as *BARX1*, *PAX9*, *LHX8* and *DLX5*, are highly expressed in GFs. It is noteworthy that these transcription factors are also active in mesenchymal cells during tooth development. Among them, *DLX5* was found to be inactivated in PAFs derived from the adult periodontitis-affected gingival tissues. This finding implies that positional memory of development is maintained in GF, and loss of GF identity is linked to pathological activation of PAFs. Transcriptional profiling of PAFs showed that genes involved in signal transduction and immune response are up-regulated (Supplementary Table S11). Further studies are needed to elucidate which signals are linked to the loss of GF identity and acquisition of the PAF phenotype.

Of particular interest was specific activation of *DLX5* and *RUNX2* distal promoters in GFs and their inactivation in PAFs. This discovery was made possible by the CAGE technology that detects TSSs and promoter regions. It is well established that *RUNX2* acts as a master regulator for osteoblast differentiation and bone formation in cell culture and animal models^17^. In humans, mutation of *RUNX2* causes cleiocranial dysplasia and teeth abnormalities^24^. Although *RUNX2* has been implicated with the tooth development, its role in GFs and periodontitis remains underexplored.

*RUNX2* has 2 major isoforms which share a common 509-amino-acid sequence. *RUNX2* short form has a distinctive 5-amino-acid N-terminal sequence that differs from the 19-amino-acid N-terminal sequence of *RUNX2* long form. These isoforms are functionally different as revealed by the studies of specific knockout mice^25^,^26^, while both are crucial in bone development^27^. The activation of *RUNX2* distal promoter occurs during osteoblast differentiation and is necessary for maintaining the osteoblast phenotype. On the other hand, the activation of *RUNX2* proximal promoter is ubiquitous both in non-osseous mesenchymal cells and osteoblast progenitors. A recent study revealed that transducing RUNX2 short form and some defined factors could cause direct conversion of human gingival fibroblasts into functional osteoblasts^28^. Furthermore, previous reports showed that inflammatory reactions could decrease the expression levels of RUNX2 in periodontal ligament fibroblasts or osteoblasts in cellular models of periodontitis^29^, and DNA hypermeltylation was suggested to be a mechanism for RUNX2 gene suppression in periodontal fibroblasts^30^. Studies on epigenetics might help understanding the regulation of RUNX2 promoter activities in gingival fibroblasts.

*DLX5* is a homeobox transcription factor involved in bone development and fracture healing^31^. Mutation in *DLX5* might be associated with split-hand/split-foot malformation^32^. It has been reported that *DLX5* specifically transactivates *RUNX2* distal promoter in committed osteoblasts^18^. In accordance, the CAGE profiles of GFs showed that activation of *DLX5* was concomitant with that of *RUNX2* distal promoter (Figs 2 and 6). We established specific knockdown of *DLX5* and *RUNX2* long form without affecting the expression of *RUNX2* short form. As anticipated, *DLX5* knockdown led to down-regulation of *RUNX* long form whereas the expression of *RUNX2* short form was not obviously altered. Noteworthy, the genes regulated by *DLX5* and *RUNX2* long form largely overlapped, further supporting the notion that *DLX5* preferentially activates *RUNX2* distal promoter and suggesting that the action of *DLX5* is largely mediated by *RUNX2* long form (Fig. 7(d)).

Given that activation of *DLX5* and *RUNX2* distal promoter is the distinctive feature of GFs, that is lost in PAFs, genes regulated by these factors are conceivably important for the homeostasis of gingival tissues. Knockdown of these factors and subsequent transcriptome analyses revealed that these factors regulate genes involved in ECM organization including collagen (*COL14A1*, *COL15A1*, *COL5A1*, and *COL8A2*), elastin, matrilin 3, dermatopontin, and fibrillin 2.

In addition, small leucine-rich proteoglycans (SLRPs) were also found to be regulated by *DLX5* and *RUNX2* long form, such as osteomodulin (*OMD*), asporin (*ASPN*), decorin (*DCN*), and osteoglycin (*OGN*). SLRPs are a group of proteins sharing various structural and functional similarities that have multiple roles in ECM regulation^33^. They are recently recognized as important regulators of cell-matrix crosstalk, influencing a variety of biological processes such as cell proliferation, differentiation, survival, adhesion, inflammation, angiogenesis, and tumorigenesis^34^.

Asporin is found to be expressed in periodontal ligaments^35^, which can bind to TGF-β1 and inhibit its ability to induce cartilage matrix gene expression^36^,^37^. Decorin has also been reported to suppress TGF-β signaling and influence matrix organization^38^. We have previously demonstrated that TGF-β signaling is activated in PAFs and is a key mediator of gingival fibroblast-epithelial cell interaction^6^. Inhibition of TGF-β signaling clearly suppressed collagen degradation in experimental models of periodontitis. Thus, our findings in the present study provide a clue to understanding the transcriptional network underlying the enhanced TGF-β signaling in PAFs. Further studies that explore matricellular functions of SLRPs, cell-matrix interactions, and TGF-β signaling activation processes would broaden our understanding of periodontitis.

In conclusion, CAGE profiling characterized distinctive transcriptional features of GFs and PAFs. Loss of GF identity appeared to be linked to the PAF phenotype, and *DLX5*-mediated alternative promoter activation of *RUNX2* is crucial for ECM organization and homeostasis of the gingiva. Disruption of this signaling in PAFs may be involved in ECM degradation and impaired architecture of the gingiva of periodontitis. These findings provide novel insight into the molecular mechanisms how PAFs develop and contribute to progression of periodontitis.

## Material and Methods

### Cell culture

Isolation and cell culture of gingival fibroblasts from the healthy or periodontitis gingival tissues were performed as described previously^7^,^11^,^39^. To minimize the risk of cell contamination from the bone, gingival tissues were incised under direct vision of the operator carefully. Gingival tissues were obtained during periodontal surgery at Nihon University School of Dentistry, Dental Hospital, Tokyo, Japan. All patients gave written informed consent. The protocol was approved by the Ethics Committee of Ohu University and Nihon University School of Dentistry. All experiments were performed in accordance with guidelines and regulations approved by the Research Ethics Committee of Ohu University and Nihon University School of Dentistry. The details of the gingival fibroblasts used in this study are shown in Supplementary Table S1. Human fetal lung fibroblasts (HFL1 and WI38) and adult normal human lung fibroblasts (NHLF) were obtained from American Type Culture Collection (ATCC) and Lonza (Basel, Switzerland), respectively. Normal dermal fibroblasts, NB1RGB, was obtained from RIKEN BRC (Tsukuba, Japan). All fibroblasts were cultured in DMEM supplemented with 10% fetal bovine serum and 1% penicillin-streptomycin.

### Three-dimensional co-culture of gingival epithelial cells and fibroblasts

Three-dimensional co-culture of gingival epithelial cells and fibroblasts were employed as an *in vitro* model of periodontitis according to the method described previously^6^,^7^,^40^. Collagen gels used for co-culture were fixed with formalin and embedded in paraffin. Vertical sections with 4 μm thickness were stained with hematoxylin and eosin (H&E).

### CAGE

The details of CAGE library generation and clustering were described previously^10^. CAGE peaks that represent transcription start sites were defined by the decomposition-based peak identification (DPI) method and annotated to genes. CAGE peaks associated with the same gene were numbered by the FANTOM5 project, according to the number of total read counts^9^. For example, we named the CAGE peak chr6:45296049 - 45296082, + as “CAGE peak 1 at *RUNX2* gene” (p1 RUNX2) since it is the first peak in terms of total read counts within the peaks associated with *RUNX2*. Thus, alternative promoters for the same gene were ranked by their expression levels, and termed as p1, p2, and p3. CAGE data with raw read counts were obtained from the FANTOM5 Table Extraction Tool, and were analyzed using the R Bioconductor package ‘edgeR’ for differential expression analysis^41^. CAGE peaks were visualized by the ZENBU browser^19^. Gene ontology (GO) analysis of differentially expressed genes was performed with DAVID web tool^42^. Classification of non-coding RNAs was performed by the annotation of GENCODE19, miTranscriptome, and PLAR^43^.

### RNA-sequencing

Total RNA was extracted using the RNeasy Mini Kit (Qiagen, Hilden, Germany). RNA-sequencing (RNA-seq) was performed at the Genome Network Analysis Support Facility (GeNAS), RIKEN CLST, Yokohama, Japan. For preparing RNA-Seq library, rRNA depletion was performed with 1 μg total RNA using TruSeq Stranded Total RNA with Ribo-Zero GoldKit (Illumina Inc. San Diego, USA). Prepared libraries were sequenced with 2 × 100 bp paired-end reads on the Illumina HiSeq 2500 sequencer (Illumina). After quality check, raw sequence reads were mapped to the hg19 genome by Tophat (version 2.0.14)^44^, and visualized with the ZENBU browser. The dataset was deposited in the Gene Expression Omnibus database (GSE81870).

### Reverse transcription quantitative PCR

Reverse transcription quantitative PCR (RT-qPCR) was performed as previously described^45^. The quantitative expression was normalized to the transcript levels of glyceraldehyde 3-phosphate dehydrogenase (*GAPDH*). The PCR primers are listed in Supplementary Table S2.

### Microarray analyses and Gene Set Enrichment Analysis

Publicly available microarray datasets including GSE3551^12^, GSE19090^15^, and GSE22029^16^, which analyze transcriptome data of various fibroblasts derived from different organs, were used for the validation. The data of GSE19090 and GS22029 were normalized using the Robust Multi-array Average algorithm (RMA)^46^, while the processed data of GSE3551 were downloaded directly from the GEO website. The significance analysis of microarrays (SAM) in the R/Bioconductor packages was applied for comparison of gene expression between two groups^47^. Heatmap visualization was performed by the EXPANDER 7.0 software package^48^. Gene Set Enrichment Analysis (GSEA) was performed using the software from the Broad Institute GSEAP 2.0^49^ on the large periodontitis microarray dataset which contains samples of 241 periodontitis and 69 healthy gingival tissues (GSE16134)^3^.

### Loss of function study of DLX5 and RUNX2 long form

Lentivirus vectors carrying artificial microRNA sequences were constructed as previously described^50^. Four pairs of sense and antisense oligonucleotides were designed for targeting human *DLX5* and *RUNX2* long form, using BLOCK-iT™ RNAi Designer (Supplementary Table S2). The annealed oligonucleotides were ligated into the pcDNA6.2-GW/EmGFP-miR vector (Life technologies, Carlsbad, CA), subcloned into the entry plasmid pDONR221, and transferred to the lentiviral expression vector, pCSII-EF-RfA. The recombinant lentivirus was produced by 293FT cells transfected with the lentiviral expression vectors, pCMV-VSV-G-RSV-Rev, and pCAG-HIVgp, using Lipofectamine 2000 reagent (Life technologies). After 72 h, the medium was collected and 1 × 10^5^ gingival fibroblasts were infected. Efficient infection was assessed by detecting EmGFP-positive cells by fluorescence microscope.

### Gene expression profiling with cDNA microarray

Total RNA was extracted 5 d after infection of lentiviruses which carry negative control (NC) miRNA (miR) or those targeting *DLX5* (miR #2 and miR #4) and *RUNX2* long form (miR #1), using the RNeasy Mini Kit. Microarray analysis was carried out using Affymetrix GeneChip^®^ Human Genome U133 Plus 2.0 array, according to the manufacturer’s instructions. Expression values less than that of negative control probe were filtered out, and cut-off value of fold change compared to NC was set to 0.5 for down- and 2.0 for up-regulated genes. The dataset was deposited in the Gene Expression Omnibus database (GSE81870).

### Statistical Analysis

Analyses of variance or Student’s *t* test for unpaired samples was used for statistical analysis. The data are expressed as means ± standard deviation (SD), and *p* < 0.05 was considered statistically significant.

## Additional Information

**How to cite this article**: Horie, M. *et al.* Transcriptome analysis of periodontitis-associated fibroblasts by CAGE sequencing identified DLX5 and RUNX2 long variant as novel regulators involved in periodontitis. *Sci. Rep.* **6**, 33666; doi: 10.1038/srep33666 (2016).

## Supplementary Material

Supplementary Information

Supplementary Table S1

Supplementary Table S2

Supplementary Table S3

Supplementary Table S4

Supplementary Table S5

Supplementary Table S6

Supplementary Table S7

Supplementary Table S8

Supplementary Table S9

Supplementary Table S10

Supplementary Table S11

Supplementary Table S12

Supplementary Table S13

Supplementary Table S14

Supplementary Table S15

Supplementary Table S16
