# Supplementary material for: Transcriptome analysis of periodontitis-associated fibroblasts by CAGE sequencing identified DLX5 and RUNX2 long variant as novel regulators involved in periodontitis
Source: Sci Rep. 2016 Sep 20;6:33666. doi: 10.1038/srep33666 (PMC5028883; doi:10.1038/srep33666)
Supplement: Supplementary Information [file srep33666-s1.pdf]

Transcriptome analysis of periodontitis-associated fibroblasts by CAGE sequencing identified DLX5 and RUNX2 long variant as novel regulators involved in periodontitis

Masafumi Horie [1,2,3], Yoko Yamaguchi [4,5], Akira Saito [1,2,*], Takahide Nagase [1], Marina Lizio [3,6], Masayoshi Itoh [3,6,7], Hideya Kawaji [3,6,7], Timo Lassmann [3,6], Piero Carninci [3,6], Alistair R. R. Forrest [3,6,8], Yoshihide Hayashizaki [6,7], Tatsuo Suzutani [9], Kai Kappert [10], Patrick Micke [11], and Mitsuhiro Ohshima [12,*]

1. Department of Respiratory Medicine, Graduate School of Medicine, The University of Tokyo, 7-3-1 Hongo, Bunkyo-ku, Tokyo 113-0033, Japan
2. Division for Health Service Promotion, The University of Tokyo, 7-3-1 Hongo, Bunkyo-ku, Tokyo 113-0033, Japan
3. Division of Genomic Technologies (DGT), RIKEN Center for Life Science Technologies, 1-7-22 Suehiro-cho, Tsurumi-ku, Yokohama, Kanagawa 230-0045, Japan
4. Department of Biochemistry, Nihon University School of Dentistry, 1-8-13 Kanda-Surugadai, Chiyoda-ku, Tokyo 101-8310, Japan
5. Division of Functional Morphology Dental Research Center Nihon University School of Dentistry, 1-8-13 Kanda-Surugadai, Chiyoda-ku, Tokyo 101-8310, Japan
6. Omics Science Center, RIKEN Yokohama Institute, 1-7-22 Suehiro-cho, Tsurumi-ku, Yokohama, Kanagawa 230-0045, Japan

7. RIKEN Preventive Medicine and Diagnosis Innovation Program, 2-1 Hirosawa, Wako, Saitama 351-0198, Japan

8. Harry Perkins Institute of Medical Research, QEII Medical Centre and Centre for Medical Research, the University of Western Australia, Nedlands, Western Australia, Australia

9. Department of Microbiology, Fukushima Medical University School of Medicine, 1 Hikariga-oka, Fukushima, Fukushima 960-1295, Japan

10. Institute of Laboratory Medicine, Clinical Chemistry and Pathobiochemistry, Center for Cardiovascular Research (CCR), Charité-University Medicine Berlin, Berlin, Germany

11. Department of Immunology, Genetics and Pathology, Akademiska Sjukhuset, Uppsala University, 751 85 Uppsala, Sweden

12. Department of Biochemistry, Ohu University School of Pharmaceutical Sciences, Misumido 31–1, Tomitamachi, Koriyama, Fukushima 963–8611, Japan

***Correspondence:**

Mitsuhiro Ohshima

Department of Biochemistry, Ohu University School of Pharmaceutical Sciences

Misumido 31–1, Tomitamachi, Koriyama, Fukushima 963–8611, Japan

E-mail: m-ohshima@pha.ohu-u.ac.jp

Phone/ FAX: +81-24-932-9149

Akira Saito

Department of Respiratory Medicine, Graduate School of Medicine, The University of Tokyo, 7-3-1 Hongo, Bunkyo-ku, Tokyo 113-0033, Japan

E-mail: asaitou-tky@umin.ac.jp

Phone: +81-3-3815-5411

FAX: +81-3-3815-5954

Supplementary Figure S1

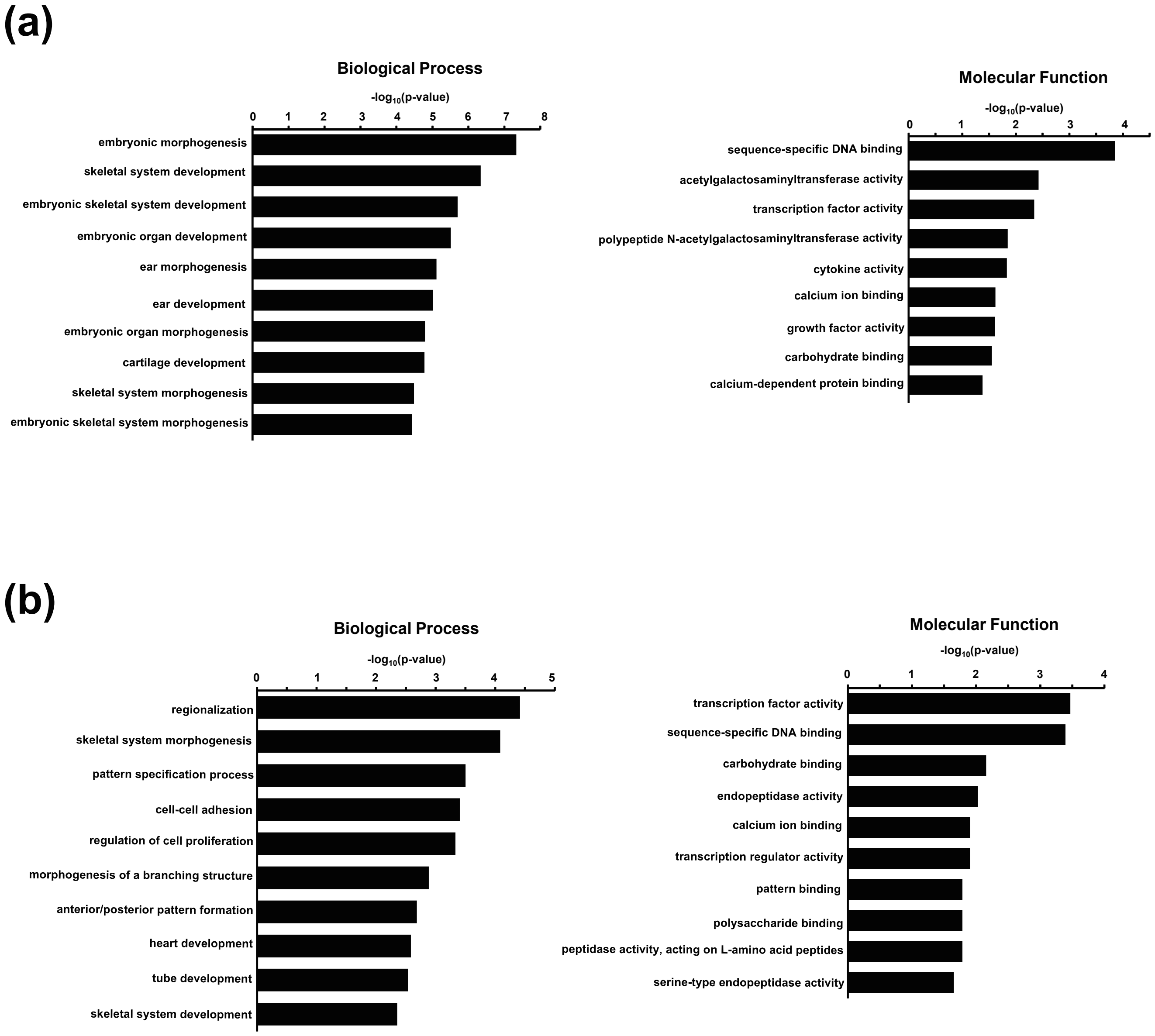

(a) Significant gene ontology (GO) terms sorted by p-value using 195 genes with higher expression in GFs. Enriched GO terms for biological process (left) and molecular function (right) are shown. Values indicate –log10 (p-value). (b) Significant gene ontology (GO) terms sorted by p-value using 109 genes with lower expression in GFs. Enriched GO terms for biological process (left) and molecular function (right) are shown. Values indicate –log10 (p-value).

Supplementary Figure S2

(a)

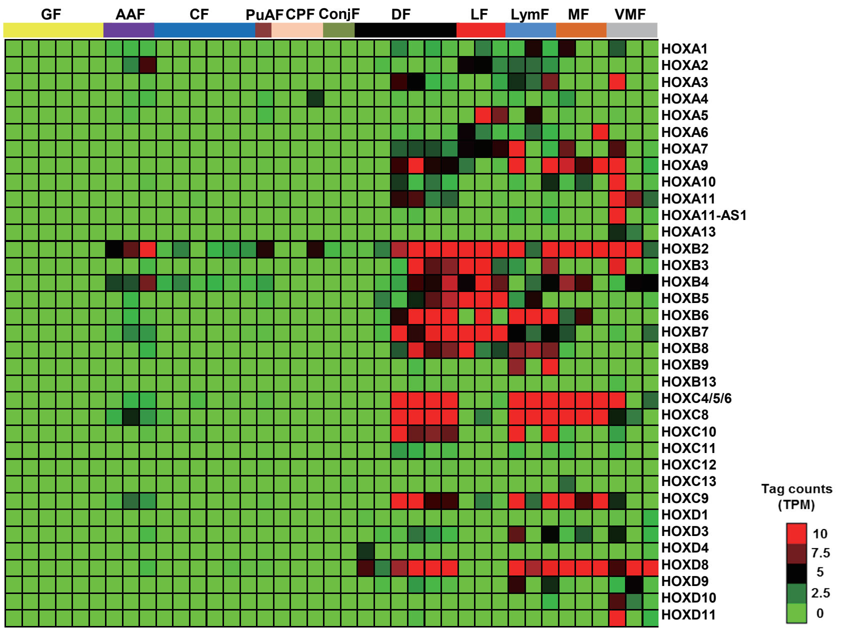

(b)

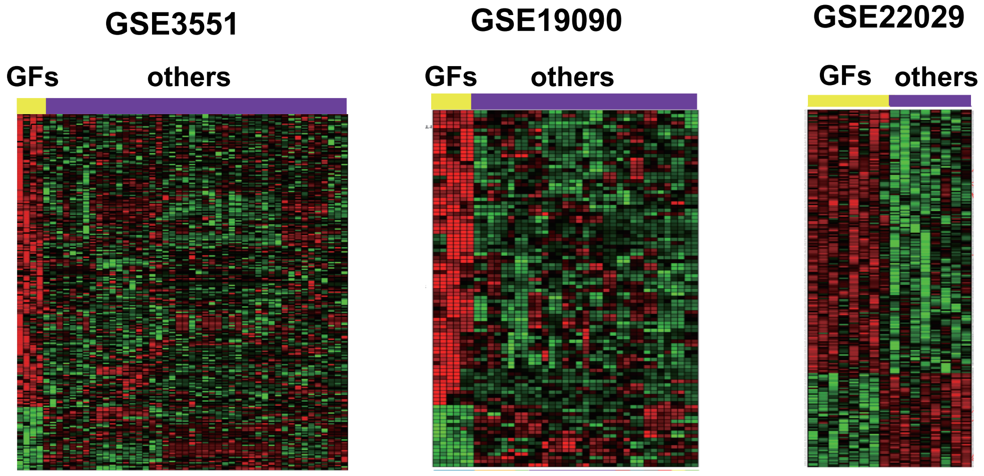

(a) Heatmap showing p1 promoter expression levels of HOX gene clusters in various fibroblasts. Red to green color gradient of heatmap represents the relative gene expression levels determined by tags per million (TPM) in GFs (yellow), AAFs (purple), CFs (deep blue), PuAF (brown), CPFs (beige), ConjFs (green), DFs (black), LFs (red), LymFs (light blue), MFs (orange), and VMFs (grey) are shown.
The details of fibroblasts are shown in Supplementary Table S1. (b) Heatmap of the top 150 genes with significantly differential expression between GFs (yellow) and other fibroblasts (purple) after sorting by FDR in GSE3551 (left, 4 GFs vs 46 other fibroblasts), GSE19090 (middle, 6 GFs vs 33 other fibroblasts), and GSE22029 (right, 8 GFs vs 8 dermal fibroblasts) microarray datasets. Red to green color gradient of heatmap represents the relative gene expression levels.

Supplementary Figure S3

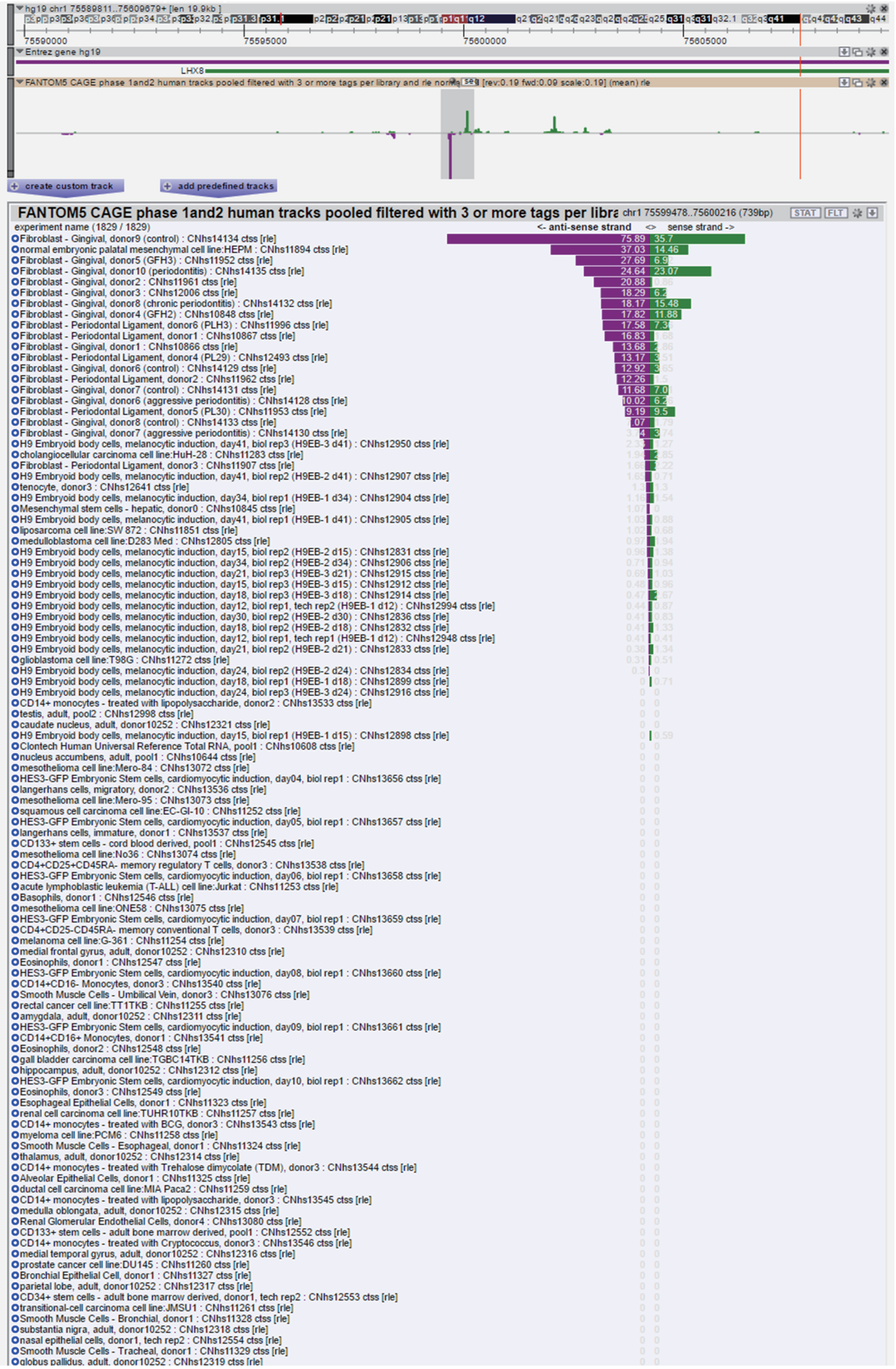

The ZENBU browser view of CAGE peaks that indicate LHX8 promoter and neighboring long non-coding RNA (lncRNA) transcribed on the reverse strand. Green and purple bars indicate forward and reverse strands, respectively. The transcript levels in different cell types are sorted by the expression values of reverse strand. Note that the cells which express LHX8 and lnc-LHX8 are almost exclusively gingival and periodontal ligament fibroblasts.

Supplementary Figure S4

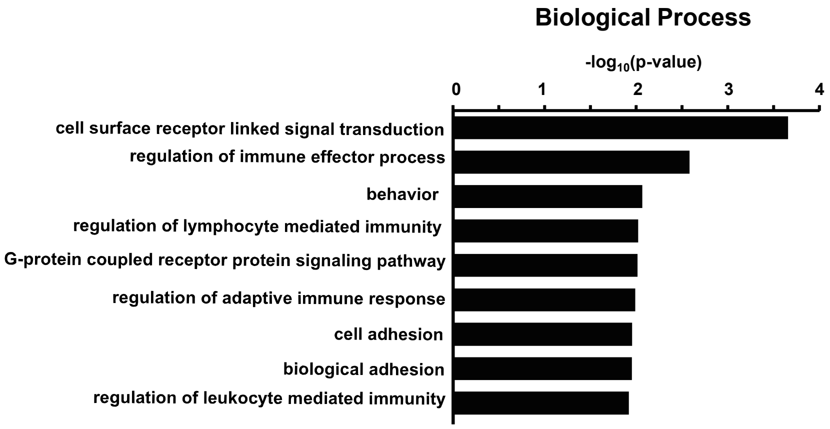

Significant GO terms sorted by p-value using 48 up-regulated genes in PAFs. Enriched GO terms for biological process are shown. Values indicate –log10 (p-value).

Supplementary Figure S5

(a)

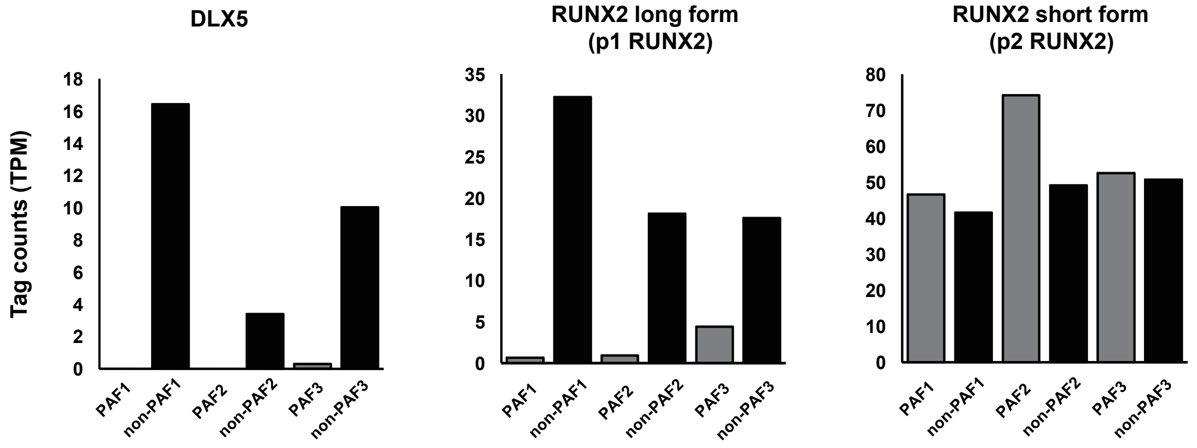

(b)

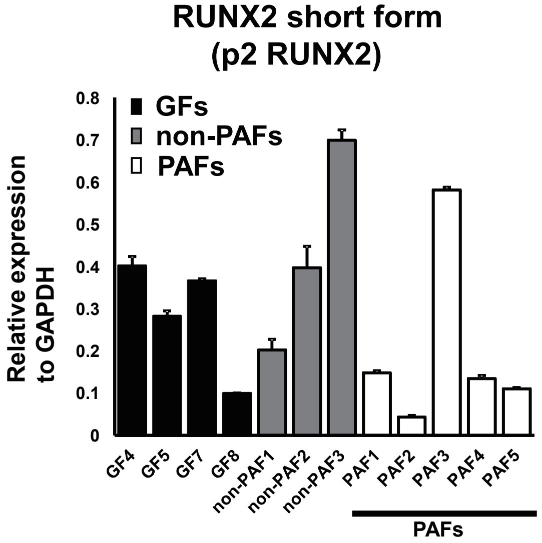

(a) CAGE tag counts (TPM: tags per million) of p1 DLX5, p1 RUNX2, and p2 RUNX2 in 3 patient-matched PAFs and non-PAFs. (b) RT-qPCR for RUNX2 short form transcribed from p2 RUNX2 promoter. The expression levels were examined in 4 control GFs (GF4, GF5, GF7, and GF8), 3 patient-matched non-PAFs (non-PAF1, non-PAF2, and non-PAF3) and PAFs (PAF1, PAF2, and PAF3), and 2 additional PAFs (PAF4 and PAF5). Each expression was normalized to that of GAPDH. Bars represent mean ± SD.

Supplementary Figure S6

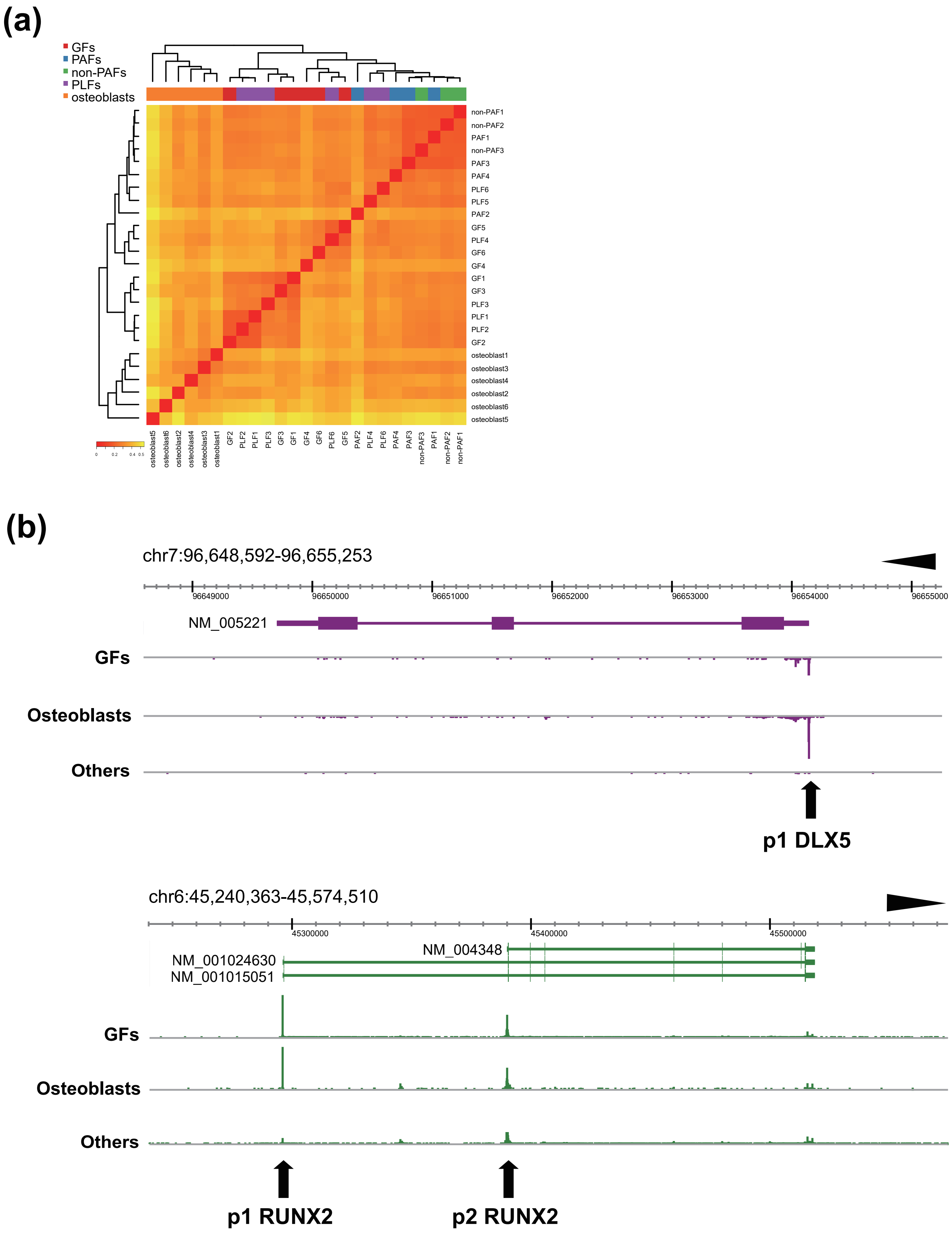

(a) Hierarchical clustering analysis with all DPIs of 4 PAFs, 3 non-PAFs, 6 control GFs, 6 PLFs, and 6 osteoblasts by Ward method. CAGE data of these cells were extracted from the FANTOM5 database. Red to yellow color gradient of heatmap represents the degree of correlation of the indicated cell pair. Note that all osteoblasts are grouped into the same cluster distinct from other cell types.
(b) Upper: CAGE peaks of 6 GFs, 6 osteoblasts, and 33 other fibroblasts visualized by the ZENBU browser. Genomic coordinate and transcript of DLX5 registered in RefSeq (NM_005221) are shown on the top. CAGE peak for p1 DLX5 is prominent in GFs and osteoblasts. Lower: CAGE peaks of 6 GFs, 6 osteoblasts, and 33 other fibroblasts visualized by the ZENBU browser. Genomic coordinate and 3 protein coding transcript variants of RUNX2 registered in RefSeq (NM_004348, NM_001024630, and NM_001015051) are shown on the top. CAGE peaks for p1 RUNX2 is detected in GFs and osteoblasts while it is absent in other fibroblasts.
